# Responsible gambling among older adults: a qualitative exploration

**DOI:** 10.1186/s12888-017-1282-6

**Published:** 2017-04-04

**Authors:** Mythily Subramaniam, Pratika Satghare, Janhavi A. Vaingankar, Louisa Picco, Colette J. Browning, Siow Ann Chong, Shane A. Thomas

**Affiliations:** 1 Research Division, Institute of Mental Health, Buangkok Green Medical Park, 10 Buangkok View, Singapore, 539747 Singapore; 2 International Primary Health Care Research Institute, Shenzhen, China; 3 Monash University, Melbourne, Victoria Australia

**Keywords:** Gambling, Limit setting, Self-exclusion, Help-seeking, Family intervention

## Abstract

**Background:**

Responsible gambling (RG) is defined as gambling for pleasure and entertainment but with an awareness of the likelihood of losing, an understanding of the associated risks and the ability to exercise control over one’s gambling activity. The current study describes a qualitative approach to explore RG among older adults (aged 60 years and above) in Singapore and reports on the cognitive and behavioural strategies employed by them to regulate their gambling.

**Methods:**

Inclusion criteria included Singapore residents aged 60 years and above, who could speak in English, Chinese, Malay or Tamil and were current or past regular gamblers. Participants were recruited using a combination of network and purposive sampling. Socio-demographic information on age, age of onset of gambling, gender, ethnicity, marital status, education and employment was collected. The South Oaks Gambling Screen (SOGS) was used to collect information on gambling activities and problems associated with gambling behaviour. Qualitative interviews were conducted with 25 older adults (60 years and above) who currently gambled. The data was analyzed using thematic network analysis.

**Results:**

This global theme of RG comprised two organising themes: self –developed strategies to limit gambling related harm and family interventions to reduce gambling harm. The basic themes included delayed gratification, perception of futility of gambling, setting limits, maintaining balance, help-seeking and awareness of disordered gambling in self or in others. Family interventions included pleading and threatening, compelling help-seeking as well as family exclusion order.

**Conclusions:**

The study highlights the significant role that families play in Asian societies in imposing RG. Education of family members both in terms of the importance of RG, and communication of the ways in which older adults can incorporate RG behaviours including the use of exclusion in specific scenarios is important.

**Electronic supplementary material:**

The online version of this article (doi:10.1186/s12888-017-1282-6) contains supplementary material, which is available to authorized users.

## Background

Gambling is a popular and widely available activity throughout the world. The majority of those who gamble are able to manage the risks associated with gambling and treat it as a leisure activity. However, in some people gambling is associated with harm to either themselves or to their families and the community. Harmful gambling has been defined as ‘any type of repetitive gambling that an individual engages in that leads to (or aggravates) recurring negative consequences such as significant financial problems, addiction, as well as physical and mental health issues’ [1]. Defining problem gambling as, ‘having difficulties limiting money and/or time spent on gambling which leads to adverse consequences for the gambler, others, or for the community’, Williams et al. [2] found that the standardized past year rate of problem gambling ranged from 0.5% to 7.6% across 202 studies conducted in various countries with an average rate of 2.3% [2].

Responsible gambling (RG) has been defined as “policies and practices designed to reduce and prevent potential negative consequences associated with gambling” [3]. Blaszczynski et al. [3] suggested that RG frameworks should incorporate government, industry, and personal responsibilities to minimize the harm related to gambling. Governments worldwide have developed and implemented legislative initiatives that are aimed at fostering RG [4, 5]. At the provider or industry level these initiatives include, but are not restricted to, display of RG signage and information, restricting under-age gambling, training of staff at gambling venues, pre-commitment and limit setting and self - exclusion [6–9]. Schaffer et al. [10] have further extended the RG framework proposed by the RENO model [10] to suggest that these principles must also extend to clinicians. Clinicians must be aware of their ethical responsibilities which include incorporating the principles of autonomy, beneficence, nonmaleficence and justice while treating patients. They also recommend that clinicians should be aware of the scientific evidence to ensure that their patients with gambling problems are treated using evidence based practices. At an individual level, the Victorian Gambling Foundation defines RG for an individual as gambling for pleasure and entertainment but with an awareness of the likelihood of losing and an understanding of the associated risks; exercising control over one’s gambling activity, and occurring in balance with other activities such that it does not cause problems or harm to themselves or others.

Studies have identified a number of RG strategies adopted by gamblers for both land based and internet gambling. Dzik [11] in his ethnographic study comprising 20 frequent casino gamblers, observed a number of RG strategies being adopted which included self- imposed wager limits and the reduction of the frequency of play by visiting the bar between bets and walking the casino floor. In a study of twelve older recreational gamblers, the cognitive and behavioral strategies adopted by them to prevent problem gambling included being fully aware of how dangerous gambling could be, avoiding cognitive distortions, setting limits and not gambling alone [12]. Wood and Griffiths [13] explored the behaviours, attitudes and motivations of “positive players” (non-problem gamblers). Positive players employed RG strategies such as setting time and wager limits, avoiding temptation by taking a predetermined amount of money to gamble, and leaving ATM cards at home. Moore et al. [14] identified five factors pertaining to gambling self-regulation strategy by using factor analysis of items generated by reviewing the literature on RG. Cognitive approaches (Factor 1) comprised items that described how gamblers tried to focus on behaviour other than gambling and to think about its negative consequences. Direct Action (Factor 2) involved gamblers reducing their gambling by executing actions such as cutting up credit cards or seeking professional assistance. Social Experience (Factor 3) consisted of items that described using gambling as a social activity or in the social context as a strategy to limit gambling. Avoidance (Factor 4) included items about avoiding gambling venues and restricting their access to money at venues, and Limit Setting (Factor 5) included items describing strategies that helped gamblers to set limits on their gambling.

Gambling is a popular social activity among older adults across many cultures [15, 16]. Older people are especially vulnerable to gambling related problems due to personal and role losses, loneliness, social isolation, declining health and fixed and lower incomes [17–20]. A recent systematic review estimated the prevalence of lifetime gambling disorder to be 0.01% to 10.6% among older adults, this prevalence, however, was found to be lower than that among young adults [21]. Singapore is a city-nation in Southeast Asia with a multi-ethnic population. The majority of Singaporeans are Chinese (74.3) followed by those of Malay (13.4) and Indian (9.1) ethnicity. About 3.2% belong to other ethnicities [22]. Singapore’s population has grown older over the years. Data suggests that the number of residents aged 65 years or older will multiply threefold to 900,000 in 2030 and that one out of every five residents will be an older adult [23]. These older adults who comprise a significant proportion of the population need and demand products and services appropriate for their age and have thus become an important consumer group. Studies have suggested that for older adults who have increased leisure time and whose physical health may limit participation in activities that they enjoyed before, gambling may provide an alternative for leisure and entertainment [17, 24]. Gambling is a popular activity in Singapore with epidemiological studies reporting that 44% of the population had gambled in the previous 12- month period [25] while 48.9% of older adults (i.e. those aged 60 years and above) reported that they had gambled at least once in their lives [26]. While prevalence of lifetime probable pathological gambling among older adults was lower than that of younger adults [26], a slightly higher prevalence of 12-month probable pathological gambling rate was found among older adults [25]. However, studies on older adult gamblers are limited and few have explored the harm caused to them or the responsible gambling strategies employed by them to prevent or minimise the harm from their perspective and lived experiences.

The current study is part of a larger qualitative study exploring gambling initiation, maintenance, harm, help-seeking and barriers to care among older adults in Singapore. The construct of RG emerged in relation to the discussion on harm as perceived by the gamblers. The current article describes the concept of RG among older adults (aged 60 years and above) in Singapore and reports on the cognitive and behavioural strategies employed by them to regulate their gambling.

## Methods

As little is known about gambling among older adults in Singapore, the decision was to use qualitative methods in an effort to “understand” the phenomenon from the perspectives of the people involved, rather than explaining it from the ‘outside’. In-depth interviews were used to gain an understanding of gambling from those older adults who gambled. In these narratives, research participants described their lived experience of gambling starting from the very first time they gambled, the progression over time and the consequences across the lifespan of the behaviour. This approach, which did not use specific pre-determined questions on RG, yielded a rich description of what older adults perceived as RG without biasing them in any way.

### Study sample

Participants were recruited using a combination of network and purposive sampling. Inclusion criteria included Singapore residents (citizens or permanent residents) aged 60 years and above, who could speak in English, Chinese, Malay or Tamil and were regular gamblers. Regular gamblers were defined as those who reported gambling at least weekly [27]. We included older adults who reported gambling regularly currently, or those who were regular gamblers in the past, i.e. had gambled regularly after the age of 60 years but may have cut down or were abstaining at the time of recruitment. Participants were also informed of their study responsibilities – i.e. willingness to talk of their gambling and that the interview would be audio-recorded. Recruitment was carried out from gambling venues like lottery outlets. Additionally, counselors and clinicians providing treatment to those with gambling disorders at the National Addiction Management Services in Singapore were informed about the study and were requested to refer suitable clients who met the inclusion criteria. A snowball sampling approach was also used by requesting participants to refer anyone they knew who met the inclusion criteria for the study. Efforts were made to ensure sample diversity in terms of gender, ethnicity and severity of gambling problems (i.e. participants who were recreational gamblers as well as those who met criteria for disordered gambling were recruited).

Older adults who were interested in participating in the study were requested to contact the first author (MS) through study information flyers. An initial screening was done over the telephone to ensure that the caller met the inclusion criteria. The callers were also asked questions to confirm that they were regular gamblers [27] and an appointment was scheduled for the face-to-face interview. Qualitative interviews were conducted with 25 older adults (60 years and above) who currently gambled. All interviews were conducted by the first author who is trained in qualitative methodology.

The study was given ethical approval by the National Healthcare Group Domain Specific Review Board, Singapore. After obtaining written informed consent, each respondent was interviewed using an interview guide (enclosed as Additional file 1 – guide for in-depth interview). The main themes that were explored during the interview included the initiation of gambling, reasons for maintaining it, progression over time and lastly, help-seeking if any. The interview guide was developed following the principles of qualitative interviewing [28], extensive literature research and with the guidance of the senior author (ST). Each interview varied between 45 min to 2 h in duration. The interviews were held at a venue preferred by the respondents. All the interviews were conducted in English, were audio recorded and subsequently transcribed verbatim. All the transcripts were checked by the first author (MS) after completion of transcription to ensure quality assurance. Handwritten notes on the transcripts and field notes of emerging themes and concepts were taken throughout the research process to maximize the dependability of the data analysis [29].

Before the commencement of the qualitative interview, the respondents were self- administered the South-Oaks Gambling Screen (SOGS) [30] using a paper and pencil version of the instrument. The SOGS comprises 20 scoring questions, each requiring a ‘yes’ or ‘no’ answer. Each ‘yes’ answer is scored as one point; answers are then added up to obtain the total score. The scoring items include questions about going back another day to win back money lost, gambling more than intended, being criticized by others over gambling, feeling guilty about gambling, having difficulty stopping gambling, and losing time from work because of gambling. SOGS has been validated in the Singapore general population. The SOGS demonstrated strong internal consistency with a Cronbach’s alpha of 0.84 [31]. For the purposes of this study, respondents scoring 5 or more on the SOGS were categorized as lifetime ‘probable pathological gamblers’, those scoring 1 to 4 were categorized as ‘lifetime problem gamblers’ and those scoring 0 as ‘non-problem gamblers’.

Socio-demographic information on age, age of onset of gambling, gender, ethnicity, marital status, education and employment was also collected using a paper and pencil version of the instrument. While both the SOGS and the socio-demographic instrument were self-administered, the interviewer was available to answer any queries or provide clarifications where needed. Two of the participants had requested that the questions be read aloud to them as they found it easier to complete the survey that way.

### Research participants

The mean age of the participants was 66.2 years. The majority were male (*n* = 18), of Chinese ethnicity (*n* = 16), had secondary education (*n* = 9), were married (*n* = 20) and currently employed (*n* = 15) in a full-time or part-time job. The mean age of gambling initiation was 24.5 years. ‘Played the numbers or bet on lotteries’ (*n* = 22) was the most frequently endorsed form of gambling by the research participants, followed by ‘betting on cards’ (*n* = 16). ‘Betting on sports’ and ‘bowled/shot pool/played any other game of skill for money’ (*n* = 6) was the least frequently endorsed by the participants. Most of them (*n* = 9) had gambled about Singapore dollars (SGD) 10–100 per day. Some of them (11) reported that other family members had gambling related problems. Nine participants met criteria for probable pathological gambling (in their lifetime) using SOGS cut-off scores of 5 and above, while 10 met criteria for some problems with gambling i.e. problem gamblers (in their lifetime) (scores of 1–4) (Table 1).

**Table 1:** Characteristics of 25 older adult Gamblers

Age at time of interview	
Range	60–81 years
Mean (SD)	66.2 (6.5) years
Age of onset of gambling	
Range	9–50 years
Mean (SD)	24.5 (10.6) years
Gender	
Male	18
Female	7
Ethnicity	
Chinese	16
Malay	2
Indian	4
Others	3
Highest Education Attained	
Primary	2
Secondary	9
A Level	3
Diploma	3
University degree and Above	6
Others	2
Marital Status	
Single	2
Married	20
Divorced/Separated	1
Widowed	2
Employment	
Employed	2
Unemployed	15
Retired	6
Largest amount of money gambled in a day	
More than $1.00 up to $10.00	3
More than $10.00 up to $100.00	9
More than $100.00 up to $1000	6
More than $1000 up to $10,000	5
More than $10,000	2
South-Oaks Gambling Screen Scores (Lifetime)	
0 (No problems with gambling)	6
1–4 (Some problems with gambling)	10
5 and above (Probable pathological gambling)	9

The choice of language was left to the respondent; however, all the respondents chose to do the interview in English. Singapore is a former English colony and one of its legacies is an educational system where English was (and remains) the main medium of instruction. The Singapore population is also one of the most literate in the world and English is widely used albeit with some local idioms introduced and local modifications made. Thus, none of the respondents had any problems expressing themselves to the interviewer.

### Data analysis

The data was analyzed thematically for the purpose of providing an overall description of the predominant themes that represented the views of older gamblers. The six-step thematic network analysis methodology proposed by Stirling [32] was adopted. Thematic analysis is a widely used qualitative technique in which data are systematically searched to identify salient themes predominantly using verbatim interview transcripts. These themes are then analysed and reported with the aim of describing the phenomenon of interest. This approach can be applied across epistemological designs [33]. Thematic network analysis makes the analysis explicit by clearly describing the links between the stages of analysis i.e. the process of going from text to interpretation is clarified using various stages [32].

Codes were identified in the text by familiarization with the data through active reading and re-reading of all the interview transcripts. Key phrases were highlighted and comments were written in the margins to record preliminary thoughts. Text segments classified in each code were read and themes were extracted. In all 27 codes were identified for RG and the text segments classified under these codes were re-read. As this was being done, a record was kept of the emerging themes. These themes were further refined to ensure that they were discrete and did not overlap with each other, yet broad enough to incorporate a set of coherent ideas. This step resulted in 27 codes, being reduced to 10 basic themes (Table 2). Coding of all the transcripts was done by the first author MS. For the first five transcripts, another researcher (ST) independently coded them and the codes and themes that were identified were discussed and refined. A codebook was created based on the mutually agreed codes and subsequent coding referred to the criteria set in the codebook. Codes and themes, especially those emerging after the detailed analysis of the first five transcripts, were also discussed with two other researchers (ST and SAC) throughout the analysis.

**Table 2:** Coding framework: Responsible Gambling

	Codes	Basic Themes	Organising themes
1	Quelling the need for immediate results	Delayed Gratification	Self –developed strategies to limit gambling related harm
2	Not participating in sport betting		Family interventions to reduce gambling harm
3	Understanding of players odds in gambling activities	Perception of futility of gambling	
4	The house always wins		
5	Understanding of the built in “house-edge”		
6	One can’t make a living out of gambling		
7	Setting loss limits	Setting Limits	
8	Setting time limits		
9	Setting win limits/ quitting while one is ahead		
10	Walking away from loss		
11	Not letting losses affect you		
12	Monitoring oneself and checking themselves if they gamble more than what they normally do		
13	Having a sense of balance between spending and losses/winning	Maintaining balance	
14	Gambling is not the priority		
15	Support groups	Help-seeking	
16	Support of religious elders and prayers		
17	Self-help books		
18	Cutting down or Quitting gambling	Abstinence	
19	Self-exclusion		
20	Significant money lost in gambling by friends or relatives	Awareness of disordered gambling in others or in self	
21	Family relationships getting strained or families breaking up		
22	Media coverage or awareness of people committing suicides due to gambling losses		
23	Awareness of disordered gambling in the past in themselves		
24	Awareness of addictive nature of gambling		
25	Family members plead/ threaten the respondent to stop gambling	Pleading and threatening	
26	Family members find out the details of treatment centres and force the older adult to seek help to help them with their gambling problems.	Compel help-seeking	
27	Family members ban respondent from casino or other specific gambling venues	Family exclusion order	

The thematic network was then developed that brings together the *basic themes* (BT) [32], which are the lowest-order themes and groups them into *organizing themes*. Organizing themes were then arranged into a super-ordinate, *global theme*, which reflects a major point in the text. These basic, organizing, and global themes were then portrayed, visually, as a network. The fourth stage involved describing and exploring the networks generated, where the content of each network was described and supported by relevant quotes. Each network was summarized in the fifth stage and finally, the key conceptual findings in the summaries of each thematic network were woven together and used to answer the original research questions.

## Results

**Fig. 1:**
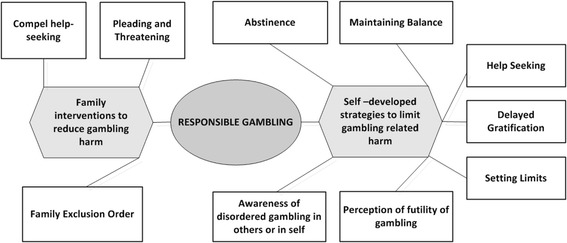
Thematic network representing responsible gambling

While discussing their gambling careers over the years, respondents mentioned the adoption of several strategies either by themselves, or imposed upon them by their family, which they felt had helped them to gamble within their means and /or exert some form of control over their gambling behaviour. This global theme of RG comprised 2 organising themes: self – developed strategies to limit gambling related harm and family interventions to reduce gambling harm (Table 2, Fig. 1). These two organizing themes are described in further detail below.

### Self –developed strategies to limit gambling related harm

#### Delayed gratification

This included quelling the need for immediate results. Respondents reported not participating in games like sports-betting where results were available immediately and where one had the ability to place bets as the game progressed thus enhancing immediate rewards. Some of them reported waiting to check on the results of the draw. Lottery-gamblers mentioned curbing their impatience to know about the results as soon as they were declared and stated that they would check for the result maybe once a month as one could claim the winnings up to 6 months after they were declared.

*I have changed. I no longer bet that much on horse-racing. In horse-racing the win or loss can trigger me to bet immediately again. Now I limit myself to it just once a week. (PG1022)*

#### Perception of futility of gambling

Other respondents reported that they knew that the “house” had an edge, thus it was difficult to beat the “system”. They knew that there was no way they could reclaim the money they had bet and that their winnings overall would be less than that what they had put in. Others stated the low likelihood of winning in these games and thus were more cautious and wary while gambling.

*Because you lose more than you win. You will never win with the machine. Because they (the operators) get the system to- always, early in the morning, - I think eleven or twelve o’clock, they already change how many swings to a strike but strike rate should not be much. You never win the jackpot. (PG 1007)*

*But after I come to understand you know, the house would always win… no matter what. They are only afraid that you win if you don’t come back, that’s the biggest problem for them you see. (PG1020)*

#### Setting limits

Many of the respondents set time or ‘money’ limits on themselves. Lottery-gamblers were very clear that they had limits on how much they would bet per day and per week. While at times these limits were broken, they were able to monitor themselves and re-set their limits again. Some of them stressed the importance of being able to walk away after losses, thereby avoiding chasing their losses. Others talked of being able to walk away with the winnings. Respondents talked about how both losses and wins could result in a heightened emotional state and in the likelihood of making bad bets.

*But I tell myself, if have- okay, don’t have also- okay. (PG 1003)*

*Alright like T (name of a friend) she doesn’t know how to cut losses she will go head on, she will charge you know and she will lose even more. (PG1010)*

*If we go with friends those days the most money I brought was 20 dollars that’s the most. Other than pay day I don’t go, when I win, for a whole month I stop. (PG1004)*

#### Maintaining balance

Some of the respondents talked about maintaining a sense of balance in terms of their behaviour i.e. curbing excessive spending, or time spent gambling, while others mentioned maintaining a sense of stability in the face of both wins and losses.

*Because some people know how to control, how to balance themselves. So, I am one of them, I like to balance it. (PG1020)*

*You have to give and take. You cannot be expecting to win all the time. (PG1008)*

*So if you strike, always keep to the same level, even though win or lose. But once you strike big, then you must stop altogether because now you are going to come back to that level again. (PG1015)*

#### Help-seeking

Many of the respondents reported the use of informal sources of help-seeking as a way to control their gambling. These included the support of self-help groups, reading self-help books, and support from religious elders and prayers.

*But thank God, there’s a church near my home. That means I can walk past this church, walk down that drive. So, sometime I just stop by that church and go in instead of proceeding on, you know. (PG1010)*

*And a.. what do you call a - priest. He advised, “You gambling is not very good. You have your parents to look after. What if you keep on gambling, and all your money's gone? Who's going to look after your parents and who's going to look after you, sir? (PG 1018)*

*I am now going for the two weeks round table talk (referring to support group), it's why we talk… probably get it off our chest. Now she (wife) is very happy, daughter's very happy. They all respect me. I take my salary, ATM card to her (wife), she does the budgeting (PG1018)*

#### Abstinence

Some of the respondents talked about cutting down or limiting the time spent on gambling if they felt that they were gambling too much. Two of the respondents talked about self-excluding themselves from casinos.

*I don’t buy 4D (a type of lottery in Singapore). Don’t think of buying 4D. Don’t see…don’t see the results in the newspaper or TV. Don’t hear…don’t hear the result in the TV or radio. Don’t want to know, your friend tells you, ‘Eh, Mr. P, your number has won.’ Say, ‘I don’t want. I…I don’t buy, I have already stopped. (PG1002)*

*Couple of times I just completely stopped you know. For a period of time, I just suddenly did not play the game… buy TOTO or big sweep (types of lottery in Singapore) or whatever. Then I stopped completely. (PG1003)*

*I just cancelled the online account. I even did not use my computer much to avoid getting online. Then I get tempted you see…because if I start once I cannot stop (PG1021)*

#### Awareness of disordered gambling in others or in self

Respondents were aware of the harms of excessive gambling. They gave anecdotal accounts of people they knew who had committed suicide, had gone bankrupt or lost their families due to excessive gambling. They also mentioned media reports that talked about the harms of gambling. Many realised the addictive potential of gambling and were either careful to control their gambling or would make the effort to reduce their gambling if they felt that they were doing it excessively.

*I know some of my friends, he gambled a lot. Then he suffered, then the children, the wife also suffered, he never cared. I don’t understand. You talk to him, it’s totally useless. Now I do not talk to him. I do tell myself…. No I am not the one going to gamble, like that. (PG1006)*

*Like, casino… I don't try because I know once I get addicted, then your whole life goes with it. As I have seen from my relations and all, I've seen some of them … house, property, everything gone. So, I know the risk of—that. (PG 1015)*

### Family interventions to reduce gambling harm

Some of the respondents highlighted the role of their families in controlling their gambling. The strategies ranged from:

#### Pleading or threatening

Some respondents talked about how family members pleaded with them or had threatened them to try to get them to cut down their gambling. The family members tried talking to the older adult gambler in order to get them to understand the harms of gambling, while others pleaded and pressurized them to stop gambling. At the extreme end, family members had made threats to sever all ties with the older adult gambler if they persisted in their gambling.

*My daughter from US, she came down. She said, ‘Daddy…please stop. Try and stop.’ See…so, she keep on pressurising me to stop. So, when she came down from US, one week, so erm…I managed to stop. For one week. Only one week so for the sake of her… I managed to stop. (PG 1002)*

*So my family has told me that if I gamble again they will break all ties with me. They have threatened me that I will lose all of them if I continue like this. (PG1023)*

#### Compel help-seeking

Older adults talked about how their family members found out the details of treatment centres and compelled them to seek treatment in an effort to get them to control their gambling related losses.

*So she was not happy (with his gambling) and she took the trouble to, you know, find out from the papers. My wife then chased after me to go to the National Addiction Management Services (PG1001)*

*Yes. I'm attending the counselling session now. They (family members) insist I go for this thing (PG1024).*

#### Family exclusion orders

Family members had sought casino-exclusion orders for the older adult gambler in an attempt to limit their losses.

*Not like here, see my family banned me here and I went into-- I tried going in (to the casino). Then, the minute I show my identity card, then they know that I'm on the self-exclusion list. And then, I'm not allowed in. They would then inform my family that I tried to get in (PG1024)*

## Discussion

Respondents in the current study did not use the term ‘responsible gambling’ but they described the use of several self-developed strategies to limit their gambling within safe limits. These respondents used self-developed cognitive and behavioural control strategies to limit their gambling to levels that were non-problematic. These included setting time and money limits for their gambling activity, monitoring themselves intermittently to ensure that they were not gambling too much, help-seeking, being aware of the harms of gambling and understanding that it was not possible to make money from gambling. Respondents also tried to curb participation in games which were associated with immediacy of rewards and in extreme cases they excluded themselves from gambling venues.

Self-regulation is defined as a key adaptation that enables humans to live in cultural groups. It allows people to change their behaviour so as to conform to the expectations of others and, to the rules and morals of the group [34]. Limit setting, monitoring themselves and re-applying themselves to self-imposed limits if there is a discrepancy seen in RG are all essential components of self-regulation that allow gamblers to conform to norms [35]. On the other hand, cognitive distortions such as superstitions, illusions of control and belief in luck observed in gamblers result in failure of self-regulation. Thus, while failure of self–regulation has been proposed as a mechanism of development of problem gambling [35]; RG demonstrates regaining or strengthening of self-regulation to prevent or regulate disordered gambling. The concept of *balance* identified by the older adult gamblers is somewhat unique to this study. The concept of Yin-Yang balance originated in China but is shared by many other Asian countries. The Yin-Yang balance is defined as integration composed of contradictions – a compromise that retains basic elements of opposing perspectives [36, 37]. Thus, while gamblers rarely spoke of abstinence or completely giving up gambling they wanted to balance the behavior with their other responsibilities and they spoke of maintaining stability in times of both wins and losses.

The themes identified in the study are similar to those identified in quantitative and qualitative studies conducted elsewhere. A phenomenological framework used by Thomas et al. [38] examined accessibility and self-regulation from the gambler’s perspective. Their sample comprised 38 participants ranging in age from 18 to 69 years and belonging to different gender, age, and gambling status. The authors identified five self-regulation strategies: *Setting Limits, Maintaining Awareness, Keeping it Social, Abstinence and Help-Seeking.* Participants also mentioned externally imposed limitations – largely by friends and families in an effort to curb their gambling. While the themes of setting limits, maintaining awareness (of gambling harm and knowing about the low probability of winning), help-seeking and abstinence are similar to the current study, the theme ‘keeping it social’ did not emerge in our study. This could be due to cultural differences between this local sample of participants and Thomas’s et al.’s [38] sample drawn from Australia. Using a phenomenological–hermeneutic approach on a small sample of 12 older social, non-problem gamblers, Hagen et al. [12] reported the use of both cognitive and behavioural strategies employed by older gamblers to keep their gambling under control. Older adults felt the realization of the potential ‘dangerousness’ of gambling, the fact that the odds are against the gambler and avoiding cognitive distortions like ‘feeling lucky’ were useful cognitive strategies in maintaining their control. Behavioural strategies such as limit setting, not gambling alone and quitting while ahead were endorsed. The similarity of themes across studies suggests that RG strategies adopted by older adults in the current sample are similar to those employed by younger adults elsewhere. It is possible that the age related decline observed in gambling is a reflection of the general decline in problem behaviours which occurs with age [39] and not due to employment of more effective RG strategies by older adults.

A number of studies have reported that family members of those with gambling problems are adversely affected [40]. Dowling [41] suggests that nagging, disapproval, threats and ultimatums are largely ineffective forms of coping and are not successful in ameliorating problem gambling. However, our study suggests that family members, by constantly engaging the respondent and setting firm limits, were frequently able to convince the respondent to gamble more responsibly.

The gamblers stated that their family’s distress resulted in behaviour modification and resulted in the use of RG strategies to avoid family conflicts and financial hardships. For Asian gamblers, family plays a significant role in stopping or cutting-down on gambling. The collectivist nature of Asian families has been suggested to have a protective role as the gamblers receive support from the family and family support may also alleviate some of the impacts of the gambling [42]. Associated closely with collectivism is the concept of stigma and face-saving that extends beyond the problem gambler to the family, and while it could lead to denial and delay of help-seeking, gamblers may not want to be stigmatised in their community or by their family and hence they may refrain from gambling [42]. Thomas et al. [38] also found that disapproval and interventions from friends and family acted as effective deterrents to excessive gambling. Singapore currently allows both nomination for the exclusion of gamblers from casinos and casino visit limits by family members. Under this scheme, family members may apply to stop their immediate relatives (spouses, children, parents and siblings) from entering the casino or limit their visits if their problem gambling has caused serious harm including financial problems and relationship issues. While only three of the respondents described their family members invoking it, given the strict enforcement of the order it compelled them to abstain from casino gambling. Surprisingly, all three respondents did not express any resentment towards their family members and they realized that the order coupled with strict screening prevented them from entering casinos and thus curtailed their gambling losses.

The findings of this study should be interpreted in the light of the following limitations. The authors were unable to recruit older adult gamblers who spoke in Malay or Chinese even though translators were available. Although the first author (MS) is a native Tamil speaker, Tamil speaking older adult gamblers also did not participate in the study. Thus despite the best effort of the authors, the study was limited to English-speaking participants and therefore possibly the more educated gamblers of all ethnicities in Singapore. The study recruited mainly those who wagered money on Mahjong, lottery and horse racing. Only three of the adults recruited were regular casino gamblers and none were regular sports gamblers. Internet gambling was declared illegal in Singapore [43] during the course of the study which had made the older adult participants more reluctant to talk about this, which limited the further exploration of this mode of gambling even among those who had used the Internet to gamble. The tendency to give socially desirable responses may have biased participants’ responses and led them to self-censor their actual experiences. Lastly data on their source of recruitment i.e. whether treatment seeking, from a gambling venue or through a referral by someone (snowballing) was not collected. This limited any comparisons between treatment seeking and non-treatment seeking sample.

## Conclusions

The study is among the first that describes RG among older adults and the cognitive and behavioural strategies employed by them to regulate their gambling in an Asian setting. The study also highlights the significant role that families play in Asian societies in imposing RG. Education of family members both in terms of the importance of RG and as well in counselling the older adults in RG behaviours, and the use of exclusion in specific scenarios is important. The current study did not examine awareness and effectiveness of gambling operator initiatives to encourage RG and reduce harm; the effectiveness of family invoked exclusion orders was also not explored. These need to be examined in further studies to obtain a comprehensive picture of RG from the player and the provider’s perspective. There is also a need for longitudinal studies to determine the effectiveness of RG strategies in preventing or curbing gambling disorder.

## Additional file

Additional file 1: Guide for in-depth interview. (DOCX 23 kb)
