# Supplementary material for: Responsible gambling among older adults: a qualitative exploration
Source: BMC Psychiatry. 2017 Apr 4;17:124. doi: 10.1186/s12888-017-1282-6 (PMC5379528; doi:10.1186/s12888-017-1282-6)
Supplement: Additional file 1: — Guide for in-depth interview. (DOCX 23 kb) [file 12888_2017_1282_MOESM1_ESM.docx]

**Guide for In-Depth Interview**

|  | |
| --- | --- |
| 1. | Tell me how you first started to gamble? |
| 2. | Would you say there was anything that contributed to your gambling? |
| 3. | What was going on in your life then? What kind of person would you describe yourself at that point? |
| 4. | Did anyone influence your decision to gamble? |
| 5. | Why did you continue to gamble? |
| 6. | Can you tell me about the good and bad things that have happened in your life that you think are related to your gambling? |
| 7. | Tell me about your gambling nowadays. Why do you gamble? |
| 8. | Tell me about the kind of person you are now? Do you think you have changed since you were N (age when first started gambling) years old? |
| 9. | When you look back can you think of any significant events associated with your gambling? |
| 10. | (IF the person has talked about problems associated with gambling) - Have you sought help for the problems such as that you had mentioned to me? |
| 11. | What has been most helpful for you to deal with these problems? |
| 12. | Where do you see yourself 5 years from now? |
| Is there anything else you want to tell me? | |
| Is there anything you want to ask me? | |
